# A case of macular telangiectasia type 2 with bilateral macular holes: Imaging features and surgical management

**DOI:** 10.1097/MD.0000000000041847

**Published:** 2025-03-21

**Authors:** Yuta Usami, Eka Rahmawati Wahyuningsih, Kana Nakano, Satoshi Kuwayama, Shuntaro Ogura, Masayo Kimura, Aki Kato, Yoshio Hirano, Yuichiro Ogura, Tsutomu Yasukawa

**Affiliations:** a Department of Ophthalmology and Visual Science, Nagoya City University Graduate School of Medical Sciences, Nagoya, Japan; b Department of Ophthalmology, Gamagori Municipal Hospital, Gamagori, Japan.

**Keywords:** fluorescein angiography, full-thickness macular hole, internal limiting membrane, macular telangiectasia type 2, microperimetry, optical coherence tomography

## Abstract

**Rationale:**

A macular hole (MH) secondary to macular telangiectasia (MacTel) type 2 is generally considered a poor surgical candidate when internal limiting membrane (ILM) peeling is performed. Due to the scarcity of studies on MH in MacTel type 2 patients, the number of published studies exploring the surgical outcomes of these patients is limited.

**Patient concerns:**

In this case report, we report a case with MacTel type 2 with bilateral MHs and had a good visual prognosis after surgical treatments.

**Diagnoses:**

A 66-year-old woman was referred to our hospital for macular abnormalities in the right eye. The best-corrected visual acuity (BCVA) was 20/32 in the right eye and 20/16 in the left eye at the initial examination. Ophthalmic examination revealed decreased retinal transparency temporal to the fovea in both eyes, cystoid spaces at the macula of the right eye by optical coherence tomography, telangiectasia temporal to the fovea, and fluorescent leakage temporal to the fovea by fluorescein angiography in both eyes and the patient was diagnosed with MacTel type 2.

**Interventions:**

Since there was no tendency toward vision loss, the patient was followed up without any treatment. Four years later, the BCVA of the right eye had decreased to 20/50, and outer retinal layer atrophy at the macula had progressed, so the patient underwent cataract surgery, pars plana vitrectomy (PPV), and fluid-air exchange. Seven months after the initial surgery, an MH was formed in the right eye. Second surgery including PPV, ILM peeling, and sulfur hexafluoride gas tamponade were performed.

**Outcomes:**

Postoperatively, the MH was closed, and the BCVA improved to 20/32 at 1 year, and 20/20 at 12 years after the surgery, respectively. Twelve years after the initial visit, an MH appeared in the left eye, and the BCVA decreased to 20/50, so cataract surgery, PPV with inverted ILM-flap technique, and sulfur hexafluoride gas tamponade were performed in the left eye. Postoperatively, the MH was closed with no recurrence for longer than 2 years after surgery, and the BCVA improved to 20/16. Microperimetry performed after the surgery showed decreased retinal sensitivity consistent with areas of retinal atrophy in both eyes.

**Lessons:**

We experienced a case of bilateral MHs associated with MacTel type 2. The right eye underwent PPV with ILM peeling and the left eye underwent PPV with an inverted ILM flap technique, both eyes had good visual prognosis. Imaging modalities and microperimetry can provide valuable information on the associated anatomical and functional changes.

## 1. Introduction

Macular telangiectasia (MacTel) type 2 is a bilateral macular disease that starts in the fifth to seventh decades of life and is characterized by reduced parafoveal gray-colored retinal transparency (retinal graying), retinal telangiectasia limited to the perifoveal region with late leakage on fluorescein angiography (FA), and neurosensory atrophy.^[1–3]^ The Beaver Dam Eye Study (cohort: 4790 people, 43–86 years of age) reported a prevalence of 0.1% in the age group analyzed based on grading from stereoscopic mydriatic fundus photographs.^[4]^ Retinal damage occurs from the temporal paracentral macula, with associated nasal scotomas. This may later involve most of the telangiectatic vessels area, including the center of the fovea.^[5]^ Consequently, visual dysfunction primarily affects patients’ reading ability, but later, more widespread loss of visual acuity (VA) may occur.^[1,5]^

The precise mechanism underlying the presence of MacTel type 2 remains controversial.^[3,6]^ The pathogenesis of MacTel type 2 may be attributed to the primary role of the retinal capillaries.^[6]^ Altered capillary walls are associated with metabolic alterations and increased endothelial permeability, inciting chronic nutritional damage to the retina, particularly to Müller cells. Müller cell dysfunction is associated with atrophy and disorganization of the outer retina, including photoreceptors, which causes gradual visual loss.^[6,7]^ FA is the gold standard for MacTel type 2 diagnosis. Spectral-domain optical coherence tomography (OCT) and OCT angiography have proven useful as adjunct imaging modalities for diagnosing MacTel type 2.^[1,8,9]^ True lamellar or full-thickness macular hole (MH) is uncommon in MacTel type 2 but have been reported.^[1,6,10]^

A MH secondary to MacTel type 2 is generally considered a poor surgical candidate, with a surgical closure rate of 30% when internal limiting membrane (ILM) peeling is performed.^[8,11]^ Due to the scarcity of studies on MH in MacTel type 2 patients, the number of published studies exploring the surgical outcomes of these patients is limited.^[12]^ Here, we report a case of a MacTel type 2 patient with bilateral macular holes (MHs) that were successfully treated with pars plana vitrectomy (PPV) and ILM peeling in the right eye, and PPV and an inverted ILM-flap technique in the left eye with sulfur hexafluoride (SF_6_) gas tamponade, and the patient could be followed-up with fundus autofluorescence (FAF) and microperimetry for longer than 2 years.

## 2. Case report

**Figure 1.**
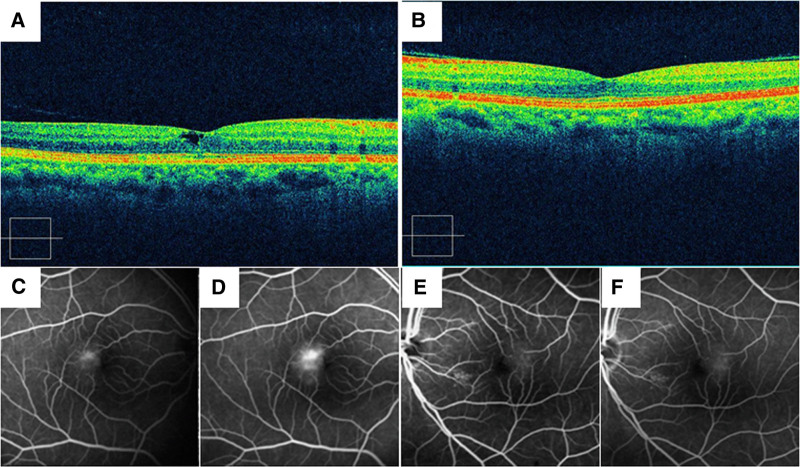
Initial findings from a 66-yr-old woman diagnosed with macular telangiectasia type 2. (A) OCT image of the right eye. A tiny retinal cavity is seen at the macula. (B) OCT image of the left eye. (C) FA (early phase) of the right eye. (D) FA (late phase) of the right eye. Hyperfluorescent spots (leakage) are seen temporal to the fovea. (E) FA (early phase) of the left eye. (F) FA (late phase) of the left eye. Fain hyperfluorescent spots are seen temporal to the fovea. FA = fluorescein angiogram, OCT = optical coherence tomography.

A 66-year-old woman who presented to an ophthalmologist with complaints of metamorphopsia in her right eye. The patient was referred to our hospital for comprehensive eye examination, and her medical history revealed diabetes mellitus. There was no notable ocular or family history. Ophthalmic examination revealed that the best-corrected visual acuity (BCVA) was 20/32 in the right eye and 20/16 in the left eye. The intraocular pressures of both eyes were normal. There were no abnormal findings notable in the anterior segments and the immature cataracts in both eyes were observed. Fundus examination revealed reduced parafoveal gray-colored retinal transparency temporal to the fovea in both eyes. OCT image across the macula revealed a hypo-reflective inner and outer retinal cavity at the foveal center with the overlying ILM drape (Fig. 1A) of the right eye. There were no apparent abnormalities in the retinal layer of the left eye, except for anterior hyaloid attachment both nasal and temporal to the macula (Fig. 1B). FA revealed hyperfluorescent spots from telangiectatic vessels situated temporal to the fovea in both eyes (Fig. 1C–F). Based on these clinical findings, the diagnosis of bilateral MacTel type 2 was confirmed. Thereafter, there was no change in the BCVA or ocular findings in the right eye, so the patient was followed-up without any treatments.

**Figure 2.**
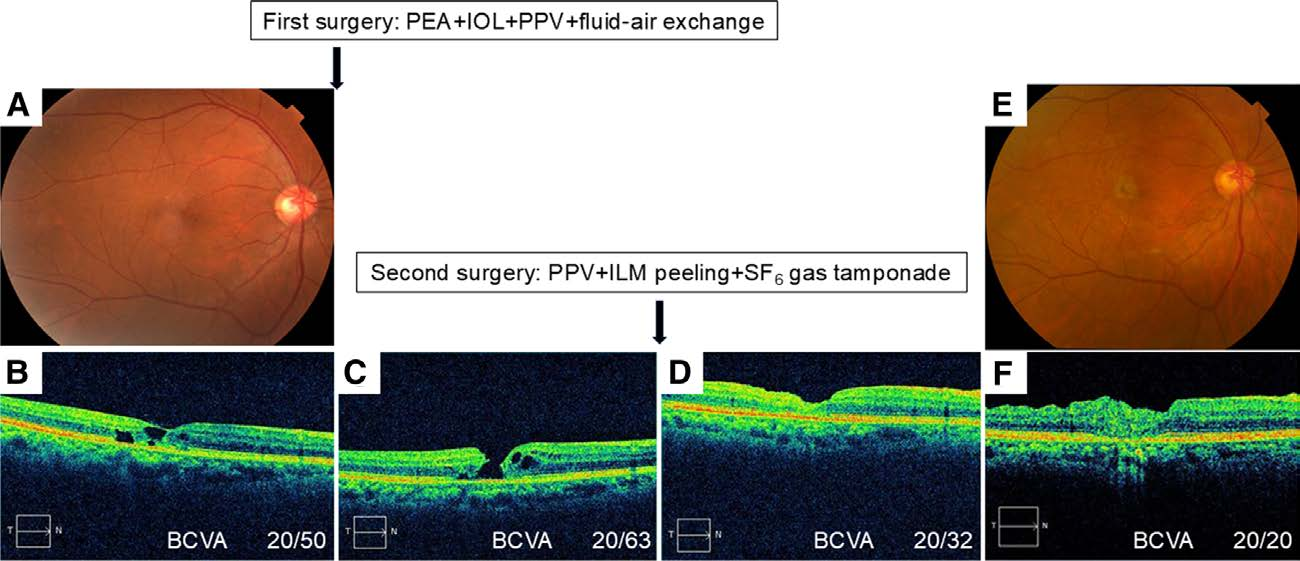
Preoperative and postoperative findings of the right eye. (A) Preoperative color fundus photograph. Gray-colored retina is observed temporal to the fovea. (B) Preoperative OCT image. Multiple retinal defects in both the inner and outer retinal layers. (C) OCT image 7 months after initial surgery. A full-thickness MH is observed. (D) OCT image 1 month after second surgery. The MH is completely closed. (E) Fundus photograph 12 yr after the second surgery. Grayish retina is seen temporal to the fovea. (F) OCT image 12 yr after second surgery. The MH remained closed, but the disorganization of inner and outer retinal layer is seen temporal to the fovea. BCVA = best-corrected visual acuity, ILM = internal limiting membrane, IOL = intraocular lens, MH = macular hole, OCT = optical coherence tomography, PEA = phacoemulsification and aspiration, PPV = pars plana vitrectomy, SF_6_ = sulfur hexafluoride.

Four years later after the initial visit, the BCVA in the right eye was further impaired to 20/50 and metamorphopsia worsened. Fundus examination showed gray-colored retina temporal to the fovea (Fig. 2A). OCT image showed multiple hypo-reflective cavities and atrophy of outer retinal layers (Fig. 2B). The patient underwent cataract surgery and PPV with fluid-air exchange in the right eye. Seven months after the initial surgery, the patient had a full-thickness MH in the right eye (Fig. 2C). The patient underwent a second surgery with PPV and ILM peeling with SF_6_ gas tamponade. The MH was closed at month 1 postoperatively (Fig. 2D). Twelve years after the surgery, the retinal atrophy temporal to the fovea had progressed (Fig. 2E), but the MH remained closed (Fig. 2F). The inner and outer retinal structures temporal to the fovea, where retinal atrophy was seen, were disrupted (Fig. 2F). The BCVA of the right eye was slightly recovered to 20/32 at 1 year and improved to 20/20 12 years after the surgery.

**Figure 3.**
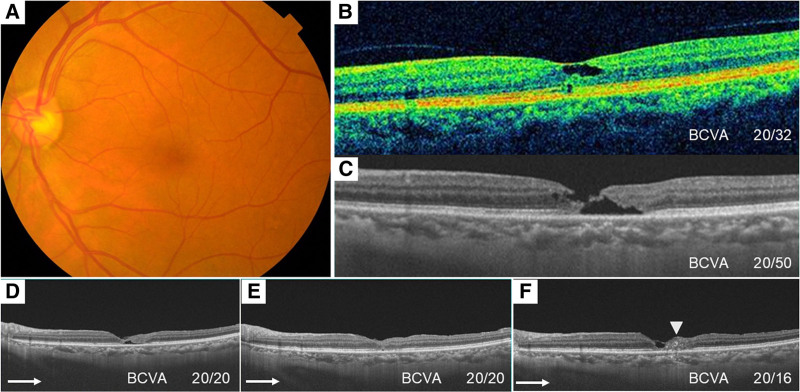
Preoperative and postoperative findings of the left eye. (A) Fundus photograph 7 yr after the initial visit. (B) OCT image 7 yr after the initial visit. Cystoid space at the macula is observed. (C) OCT image 12 yr after the initial visit. Retinal cavities in both inner and outer retinal layers are enlarged and a full-thickness MH is observed. (D) OCT image 1 month after the surgery. The MH is almost closed, but the outer retinal cavity is observed. (E) OCT image 6 months after the surgery. The MH is completely closed. (F) OCT image 22 months after the surgery. The MH is still closed, but the outer retinal layer has thinned, and the retinal structure temporal to the fovea is disorganized, and hyperreflective materials (white arrowhead) are seen. BCVA = best-corrected visual acuity, MH = macular hole, OCT = optical coherence tomography.

Seven years after the initial visit, the patient experienced a decreased vision in the left eye. The BCVA of the left eye was 20/32. Fundus examination showed gray-colored retina temporal to the fovea (Fig. 3A). A scan using OCT revealed an inner retinal cavity within the macular area (Fig. 3B). Five years later (12 years after the initial visit), the patient complained of blurred vision in the left eye, and the BCVA was decreased to 20/50. The OCT image revealed a full-thickness MH (Fig. 3C). The patient underwent cataract surgery and PPV with an inverted ILM-flap technique and fluid-air exchange with SF_6_ gas tamponade. Following the procedure, the MH was almost closed at 1 month after the surgery, although there was tiny cavity in the outer retina (Fig. 3D), and the MH was fully closed at the 6-month postoperatively (Fig. 3E) and the BCVA improved to 20/20. Twenty-two months after the surgery, the BCVA improved to 20/16, but the outer retina at the center had thinned, and disorganization of the inner and outer retinal layers and hyperreflective materials were seen temporal to the fovea (Fig. 3F).

**Figure 4.**
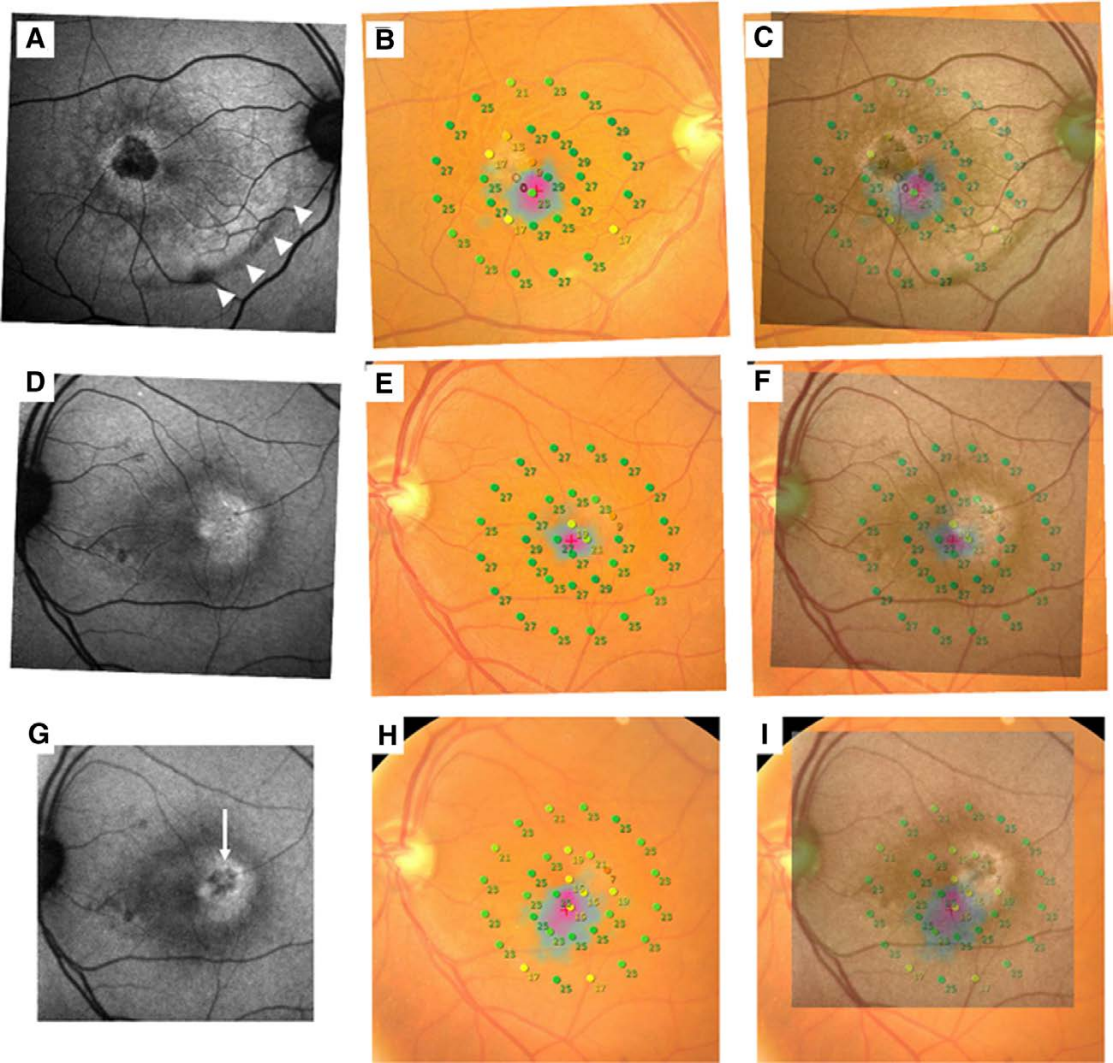
Fundus autofluorescence (FAF) and microperimetry of both eyes. (A) FAF image of the right eye 12 yr after the surgeries. Hypo-autofluorescent area is observed in the center of the increased FAF area temporal to the fovea. Another hypo-autofluorescent line (white arrowheads) indicates the previous ILM peeled area. (B) Microperimetry of the right eye 12 yr after the first surgery. (C) Superimposed image of FAF (A) and microperimetry (B). The retinal sensitivities located in the hypo-autofluorescent area are decreased. (D) FAF image of the left eye 12 months after the surgery. Hyper-autofluorescent spots are observed temporal to the fovea (E) Microperimetry of the left eye 12 months after the surgery. (F) Superimposed image of FAF (D) and microperimetry (E). (G) FAF image of the left eye 22 months after the surgery. Hypo-autofluorescent spots (white arrow) are observed in the center of the hyper-autofluorescent area. (H) Microperimetry of the left eye 22 months after the surgery. (I) Superimposed image of FAF (G) and microperimetry (H). ILM = internal limiting membrane.

In the patient, FAF and microperimetry were also evaluated (Fig. 4). In both eyes, an increased FAF area was seen temporal to the fovea, possibly due to reduced blockage by macular pigment (Fig. 4A, D, and G), and the retinal sensitivities were reduced in this area (Fig. 4B, C, E, F, H, and I). As for the right eye, the center of the increased FAF was hypo-autofluorescence due to atrophy after longer than 12 years (Fig. 4A), and the retinal sensitivities in that area were particularly reduced (Fig. 4B and C). As for the left eye, longer than 2 years after the surgery, FAF showed hypo-autofluorescence at the center of the increased FAF area, where the retinal sensitivities were also reduced (Fig. 4H and I).

## 3. Discussion

A full-thickness MH secondary to MacTel type 2 is not a frequent complication and was initially described by Olson et al,^[13]^ but the case was not treated. An MH is a prominent cause of vision loss in MacTel type 2 patients, and the standard care for MH is PPV to restore a MH and the VA.^[10,14,15]^ However, existing studies have reported that PPV in this setting has a guarded prognosis, with a closure rate of approximately 25% to 30%.^[10,12,14]^ and the outcomes for treating MHs related to MacTel type 2 are less favorable than those for treating idiopathic MHs, as there is a greater likelihood of the MH not closing or reopening.^[10,14]^

In eyes with MacTel type 2, the degeneration and atrophy of Müller cells are thought to be the cause of MH occurrence and a worse surgical closure rate compared with typical idiopathic MHs.^[10,12,14]^ Additionally, there is loss of the ellipsoid zone, development and coalescence of cystic cavities, and ultimately, dissolution of the neurosensory retina.^[15]^ Tissue defects caused by Müller cell atrophy, which may be the primary structural support of the fovea, can cause foveal structural instability. Gass^[16]^ and Koizumi et al^[17]^ speculated that Müller cell degeneration in MacTel leads to physical loss of retinal tissue, leaving a cystic space that may develop into a MH, even without significant vitreous traction.^[12,18]^ It is also thought that the tissue defect from Müller cell atrophy and lack of regeneration of the neurosensory retina may cause a lack of improvement in BCVA and result in a decrease in the rate of MH closure when surgery is performed.^[12,15]^

Since Kelly and Wendel reported^[19]^ the usefulness of PPV for treating MHs, surgical treatment for this condition has become feasible.^[19–21]^ Eckardt et al^[22]^ first reported the use of ILM peeling as a treatment for MH. Since then, the rate of MH closure has increased significantly, reaching 95%, with notable improvements in BCVA.^[23,24]^ ILM peeling removes Müller cone cells and induces trauma to the Müller cell end feet, leading to the proliferation of retinal glial cells and enhancement of MH contraction and repair.^[10,24]^ According to histological research, the process of ILM peeling is known to trigger the activation of Müller cells, resulting in the secretion of collagen, basement membrane components, and inflammatory substances. This subsequent activation of glial cell-mediated closure of MHs has been observed to occur.^[12]^

Only a few reports have addressed MacTel type 2 complicated with MH, and even fewer reports of surgical intervention in such patients exist. Rishi and Kothari first reported the surgical management of patients with an MH of MacTel type 2.^[25]^ Since that report, several reports have followed the surgical outcomes of patients treated with MH associated with MacTel type 2. However, surgical outcomes are usually unsatisfactory.^[12,14]^ A previous report by Kedarisetti showed that PPV combined with ILM peeling did not improve the anatomical or functional outcome of MacTel type 2 because the vitreous adhesion and traction may cause foveal changes.^[7]^ A global multicenter study by Park et al concluded that surgical closure of MHs improved BCVA in 57% of the eyes.^[20]^ Compared with ILM peeling alone, ILM-flap technique achieved better anatomic and functional outcomes.^[20]^ Autologous retinal transplantation could be a potential solution for refractory MHs, but the BCVA resulting from this method was not significantly improved.^[20]^

In the current study, we reported a case of Mactel type 2 complicated by bilateral MHs treated with ILM peeling in the right eye and the inverted ILM-flap technique in the left eye with successful MH closure and BCVA recovery. After the surgeries, there was no reopening of the MH, and the final BCVA was 20/20 at the 12-year follow-up in the right eye and at the 2-year follow-up in the left eye. The possibility that this may have contributed to our successful surgical outcomes is the greater extent of ILM peeling close to the major arcade vessels and our reinforcement of strict head positioning for 7 days after surgery. Previous study by Ahmad reported that ILM peeling of the major vascular arcade affected the success of surgery in the eye with MH associated with MacTel type 2.^[12]^

In 2010, the inverted ILM-flap technique reported by Michalewska et al^[26]^ emerged as a new and effective approach, particularly for the management of large, complex, and myopic MHs.^[21,23]^ It has been reported that the technique consisting of partial peeling of the ILM and placement of the flap over the hole increased the rate of complete MH closure to 98% for large idiopathic MHs (>400 μm) compared with 88% for conventional PPV and ILM peeling.^[21,26,27]^ To increase the possibility of MH closure, we performed the inverted ILM-flap technique in the left eye that might provide a structural scaffold for the proliferation and migration of activated Müller cells that promote the closure of MH-producing neurotrophic factors and basic fibroblast growth factor.^[10,21,23,27,28]^ The migration and gliosis of Müller cells can be achieved when, rather than being surrounded by vitreous fluid, they are in a semidry environment by gas replacement. To induce more intense and longer-term gliosis of Müller cells, it is important that patients remain in the prone position for a long period after inversion or transplantation of the ILM.^[28]^

Previous reports have shown that no significant change in BCVA was noted in eyes with hole closure and that there was no difference between eyes underwent PPV treatment and eyes with observation only.^[14,29]^ However, our case showed that vision improved to complete recovery (20/20 in the right eye and 20/16 in the left eye), and the MH remained closed. Sborgia et al similarly reported that vision improved to 20/20 in eyes with closure of the MH by the inverted ILM-flap technique.^[10]^ Fortunately, in this patient, the MH was closed in both eyes, and the visual prognosis was good in both eyes. However, there are several reports of MH reopening.^[8,14,15,18,19,29,30]^ Actually, the outer layer of the left eye that underwent PPV with inverted ILM-flap technique is gradually thinning. Careful monitoring of the patient’s progress is necessary to ensure that reopening of MH does not occur in the future.

In the current patient, we evaluated not only VA and OCT, but also FAF and microperimetry to assess visual function and retinal structure. FAF revealed an increased FAF area temporal to the fovea in both eyes, and decreased retinal sensitivity in this area. As for the right eye, FAF revealed hypo-autofluorescent area temporal to the fovea, which was located inside of the increased FAF area, and the retinal sensitivities were particularly reduced in that area. MacTel type 2 is a disease of Müller cells degeneration,^[16,17]^ and long-term follow-up is necessary to confirm the progression of atrophy. These findings in the right eye were due to postoperative for longer than 12 years, and similar change was observed in the left eye (Fig. 4G). Long-term continuous follow-up is still needed.

The current report has several limitations. The first limitation is that we studied only 1 patient. This retinal disorder is very rare, and it is difficult to examine many cases. The second limitation is that we performed cataract surgery combined with PPV and an inverted ILM-flap. The visual improvement observed in our patient may have been due to the phacoemulsification component of the surgery. However, anatomical closure of the MH was still achieved, which may also be associated with vision improvement in eyes with MH associated with MacTel type 2.^[14]^

Surgical treatment seems to be 1 of the options for MH closure and BCVA restoration. However, this retinal disorder is rare, and only a few cases have been examined. Additional investigations are required to determine the correlation between anatomical success and BCVA in a larger number of patients with MHs associated with MacTel type 2.^[14,31]^ The strengths of our case include that the retinal disorder occurred in both eyes, and the prior affected eye could be observed longer than 12 years after the surgeries. We were also able to observe the subsequent course of both eyes that had undergone different surgeries using OCT, FA, and FAF imaging, as well as microperimetry. Retinal imaging and microperimetry could provide additional tools for evaluating both anatomical and retinal functional changes.

## Acknowledgments

All authors have participated in clinical care of the patient and worked on the literature review and modification of our manuscript. We appreciate all the efforts contributed by all the authors of this article.

## Author contributions

**Conceptualization:** Yoshio Hirano.

**Data curation:** Yuta Usami, Yoshio Hirano.

**Formal analysis:** Yoshio Hirano.

**Investigation:** Yuta Usami, Masayo Kimura, Yoshio Hirano.

**Methodology:** Masayo Kimura, Yuichiro Ogura.

**Project administration:** Yoshio Hirano.

**Resources:** Yuta Usami, Masayo Kimura.

**Supervision:** Tsutomu Yasukawa.

**Validation:** Kana Nakano, Satoshi Kuwayama, Shuntaro Ogura, Aki Kato.

**Visualization:** Yoshio Hirano.

**Writing – original draft:** Yuta Usami, Eka Rahmawati Wahyuningsih.

**Writing – review & editing:** Yoshio Hirano.
